# Injectable Biomimetic Hydrogels as Tools for Efficient T Cell Expansion and Delivery

**DOI:** 10.3389/fimmu.2018.02798

**Published:** 2018-11-28

**Authors:** Jorieke Weiden, Dion Voerman, Yusuf Dölen, Rajat K. Das, Anne van Duffelen, Roel Hammink, Loek J. Eggermont, Alan E. Rowan, Jurjen Tel, Carl G. Figdor

**Affiliations:** ^1^Department of Tumor Immunology, Oncode Institute, Radboud Institute for Molecular Life Sciences, Radboud University Medical Center, Nijmegen, Netherlands; ^2^Institute for Molecules and Materials, Radboud University, Nijmegen, Netherlands; ^3^Materials Science Centre, Indian Institute of Technology Kharagpur, Kharagpur, India; ^4^Department of Biomedical Engineering, Laboratory of Immunoengineering, Eindhoven University of Technology, Eindhoven, Netherlands; ^5^Institute for Complex Molecular Systems, Eindhoven University of Technology, Eindhoven, Netherlands

**Keywords:** adoptive T cell transfer, biomaterial-based scaffold, polyisocyanopeptide hydrogel, 3D culture, injectable, T cells

## Abstract

Biomaterial-based scaffolds are promising tools for controlled immunomodulation. They can be applied as three dimensional (3D) culture systems *in vitro*, whereas *in vivo* they may be used to dictate cellular localization and exert spatiotemporal control over cues presented to the immune system. As such, scaffolds can be exploited to enhance the efficacy of cancer immunotherapies such as adoptive T cell transfer, in which localization and persistence of tumor-specific T cells dictates treatment outcome. Biomimetic polyisocyanopeptide (PIC) hydrogels are polymeric scaffolds with beneficial characteristics as they display reversible thermally-induced gelation at temperatures above 16°C, which allows for their minimally invasive delivery via injection. Moreover, incorporation of azide-terminated monomers introduces functional handles that can be exploited to include immune cell-modulating cues. Here, we explore the potential of synthetic PIC hydrogels to promote the *in vitro* expansion and *in vivo* local delivery of pre-activated T cells. We found that PIC hydrogels support the survival and vigorous expansion of pre-stimulated T cells *in vitro* even at high cell densities, highlighting their potential as 3D culture systems for efficient expansion of T cells for their adoptive transfer. In particular, the reversible thermo-sensitive behavior of the PIC scaffolds favors straightforward recovery of cells. PIC hydrogels that were injected subcutaneously gelated instantly *in vivo*, after which a confined 3D structure was formed that remained localized for at least 4 weeks. Importantly, we noticed no signs of inflammation, indicating that PIC hydrogels are non-immunogenic. Cells co-delivered with PIC polymers were encapsulated within the scaffold *in vivo*. Cells egressed gradually from the PIC gel and migrated into distant organs. This confirms that PIC hydrogels can be used to locally deliver cells within a supportive environment. These results demonstrate that PIC hydrogels are highly promising for both the *in vitro* expansion and *in vivo* delivery of pre-activated T cells. Covalent attachment of biomolecules onto azide-functionalized PIC polymers provides the opportunity to steer the phenotype, survival or functional response of the adoptively transferred cells. As such, PIC hydrogels can be used as valuable tools to improve current adoptive T cell therapy strategies.

## Introduction

Scaffolds produced from a variety of biomaterials are now widely applied as engineered microenvironments or delivery vehicles in biomedical applications. These biomaterial-based scaffolds can be used as three-dimensional (3D) culture systems *in vitro* to more faithfully recapitulate the complex set of cues that cells receive in the body (1). Alternatively, biomaterial-based scaffolds can be applied *in vivo* as delivery vehicles of bioactive molecules or cells, since they can exert spatiotemporal control over the release of bioactive molecules (2, 3) and dictate cellular localization (4, 5). Precisely these characteristics can be highly valuable for the field of immunoengineering to benefit cancer immunotherapy, as scaffolds can be applied as tools to induce strong and durable anti-cancer immune responses (6, 7).

Biomaterial-based scaffolds are able to overcome several limitations associated with current cancer immunotherapeutic strategies and thereby enhance efficacy and reduce treatment-related toxicity. For instance, scaffolds have been used for efficient cancer vaccination by recruiting dendritic cells (DCs) toward a depot of tumor antigens and adjuvants in the context of a local 3D environment in the body, which obviates the need for time-intensive *ex vivo* DC culturing protocols (8–10). Alternatively, toxicity associated with systemic immune checkpoint blockade can be reduced by the local and sustained release of anti-programmed death ligand 1 (PD-L1) and chemotherapy from scaffolds (11). By acting as molecular and cellular delivery vehicles with high spatiotemporal resolution, biomaterial-based scaffolds can have a clear additive value to current cancer immunotherapeutic strategies.

The ability to control the 3D environment and direct cellular localization can be especially beneficial to enhance the efficacy of cellular cancer immunotherapies such as adoptive T cell transfer (ACT). Adoptive transfer of T lymphocytes is aimed at eliminating tumor cells by infusing cancer patients with high numbers of autologous tumor-reactive tumor infiltrating lymphocytes (TILs). This potent strategy exploits the natural capacity of cytotoxic T cells to recognize and kill cancerous cells, and encouraging results have been reported for various solid cancer types (12–15). However, systemic injection of expanded tumor-reactive T cells results in insufficient localization of infused lymphocytes to the tumor site and a lack of *in vivo* persistence (16, 17), even though high cell quantities (typically 10^10^ cells) are administered. Moreover, for many cancer patients it is not feasible to generate these large amounts of TILs, which is one of the factors that hampers widespread application of ACT across different solid cancers types (18). Lymphodepleting conditioning of the host and co-infusion of high dose bolus IL-2 are applied to enhance the accumulation and survival of adoptively transferred cells (19), but both cause significant wide-spread toxicity (18). Thus, poor T cell persistence and functionality hamper the clinical efficacy of ACT for solid tumors (20–24), particularly since the degree of persistence of the administered lymphocytes is associated with outcome (25, 26). There is a great medical need to develop more efficient and rapid approaches for the *ex vivo* expansion of TILs and to improve the delivery and persistence of T lymphocytes. These hurdles can be overcome by making use of biomaterial-based scaffolds as efficient 3D culture systems and by dictating cellular localization by exploiting scaffolds as cellular delivery vehicles.

In this study, we explore the potential of an injectable scaffold to harbor and support the expansion of pre-activated T cells *ex vivo* and we studied the feasibility of injecting these gels *in vivo* for localized T cell delivery. We present a scaffold that consists of a polymeric hydrogel that is based on fully-synthetic tri-ethylene glycol-substituted polyisocyanopeptides (PIC). Hydrogels generally provide excellent biocompatibility due to their high water-content which facilitates rapid diffusion of nutrients and chemical cues. The PIC hydrogels are composed of a bundled network of synthetic PIC polymers (27), which have the advantage that they are well-defined and have a tunable composition. These polymers allow for incorporation of azide click-handles, which can be used to functionalize the gels with biomolecules such as integrin-binding motifs (28, 29). In particular, a unique feature of PIC hydrogels is their biomimetic strain-stiffening behavior that resembles the mechanical behavior found in natural polymers, where upon strain the stiffness of the material increases (27, 30). The PIC polymers display thermally-induced gelation at temperatures above 16°C, upon which they form into a transparent hydrogel (27, 30). This enables straightforward encapsulation of cells and importantly enables delivery of these gels *in vivo* via needle-mediated injection. The PIC gels can therefore be administered in a minimally invasive manner, which diminishes risks of complications that are associated with implantation of scaffolds.

Here, we demonstrate that PIC hydrogels support the survival and expansion of *ex vivo* cultured T lymphocytes. We furthermore describe the *in vivo* gelation of PIC polymers after injection and study its biocompatibility. Collectively, we provide evidence that PIC hydrogels are highly promising 3D scaffolds for the *in vitro* expansion and *in vivo* delivery of pre-activated T cells which puts PIC hydrogels forward as valuable tools to improve current adoptive T cell therapy strategies.

## Materials and methods

### Preparation of polyisocyanopeptide polymers and conjugation of grgds peptide

Non-functionalized and azide-functionalized tri-ethylene glycol-substituted polyisocyanopeptide (PIC) polymers were synthesized as previously described (28, 29). Briefly, non-functionalized isocyano-(D)-alanyl-(L)-alanine monomer and azide-terminated monomer mixed at a molar ratio of 1:30 were dissolved in toluene. After adding nickel percholate (Ni(ClO_4_)_2)_ as a catalyst (catalyst-to-monomer molar ratio of 1:1,000), the reaction mixture was stirred at room temperature for 72 h. The reaction product was precipitated three times from dichloromethane in di-isopropyl ether. To produce GRDGS-functionalized PIC polymers (RGD-PIC), a solution of DBCO-NHS in DMSO was mixed with GRGDS peptide dissolved in borate buffer (pH 8.4) at 2 mg/mL and stirred for 3 h at room temperature. Mass spectrometry was performed to confirm the formation of DBCO-GRGDS conjugates. DBCO-GRGDS peptide was conjugated to azide-functionalized PIC polymers via strain-promoted azide-alkyne cycloaddition at a ratio of one DBCO-GRGDS per 100 monomers (28). DBCO-sulfo-Cy5 (Jena Bioscience) at a ratio of one DBCO-sulfo-Cy5 per 5,000 monomers was conjugated to the azide-functionalized PIC polymers in a similar manner to generate fluorescent PIC polymers.

### Rheological analysis and characterization of PIC polymers and hydrogels

RGD-PIC and PIC polymers were dissolved at 3 mg/mL in *X-VIVO*-15 medium (Lonza) supplemented with 2% human serum (HS) by rotation at 4°C for 24–36 h and dissolved polymers were stored at −20°C. Bulk stiffness measurements on gels was done by rheology analysis at 37°C were performed as described (28) using temperature sweep rheology followed by time sweep experiments. Peripheral blood leukocytes or pan T cells were incorporated in the gels at a concentration of 0.5 or 1 × 10^6^ cells/mL at gel concentrations of 0.75 and 1.5 mg/mL. PIC polymers were furthermore characterized by circular dichroism at 0.1 mg/mL in PBS on a Jasco 815 CD spectrophotometer. Atomic force microscopy was performed to confirm appropriate length and molecular weight (28). In all functional experiments rheological analysis was performed to validate proper gel formation and gel bulk stiffness.

### Preparation of collagen gels

Three-dimensional collagen matrices were prepared at 1.7 mg/mL by mixing 55.5% (v/v) of pepsinized bovine type 1 collagen (3.1 mg/mL, PureCol, Advanced Biomatrix), 3.7% (v/v) 0.75% sodium bicarbonate solution (Life Technologies), 7.4% (v/v) minimum essential Eagle's medium (Sigma-Aldrich) and 33.3% (v/v) *X-VIVO*-15 medium with 2% HS containing cells at a final concentration of at a concentration of 0.5 or 1 × 10^6^ cells/mL. The matrices (final pH 7.4) were polymerized at 37°C for 30–45 min.

### Cell isolation, cell culture, and reagents

Human dendritic cells, pan T cells and natural killer (NK) cells were isolated from buffy coats obtained from healthy volunteers. This study was carried out in accordance with the recommendations of institutional guidelines. All subjects gave written informed consent in accordance with the Declaration of Helsinki. CD1c^+^ mDCs were isolated from peripheral blood mononuclear cells (PBMCs) using the CD1c (BDCA-1) DC isolation kit. Pan T cells and NK were isolated from peripheral blood leukocytes using the Pan T cell isolation kit and the NK cell isolation kit, respectively, according to manufacturer's prescription (all Miltenyi Biotec). Cell phenotype was determined using flowcytometry staining: CD11c (BD Biosciences)/CD1c (Miltenyi Biotec) for CD1c^+^ mDCs (purity>85%), CD3 (eBioscience) for pan T cells (purity >98%), CD69 and CD25 (both BD Pharmingen) for T cell activation, CD56 (BD Biosciences) for NK cells (purity >92%). Human DCs, T cells and NK cells were cultured in *X-VIVO*-15 medium supplemented with 2% HS. For NK cell culture an additional 100 IU/mL of IL-2 (Proleukin, Norvartis) was added.

### Embedding cells within PIC gels and cell viability

Human pan T cells were activated overnight using plate-immobilized αCD3 monoclonal antibodies (clone OKT3, 1 μg/mL, BioXcell) and αCD28 monoclonal antibodies (clone 9.3, 5 μg/mL, BioXcell). Non activated pan T cells, activated pan T cells, immature DCs or NK cells were incorporated within (RGD-) PIC hydrogels by mixing cold (RGD-) PIC polymers with the cells on ice at a final gel concentration of 0.75 or 1.5 mg/mL and a cell concentration of 0.5–1 × 10^6^ cells/ml unless indicated otherwise. Instantaneous gelation of the RGD-PIC gel when placed at 37°C ensured embedding of cells throughout the 3D matrix. Pan T cells were labeled with PKH-26 (PKH26 Red Fluorescent Cell Linker Kit, Sigma Aldrich) and imaged on Olympus FV1000 Confocal Laser Scanning Microscope to test distribution in the gel, and a z-stack reconstruction was made using FIJI software. For cell viability experiments, cells were simultaneously encapsulated in collagen scaffolds. After 4, 24, 48, and 72 h, cells were recovered from (RGD-) PIC gels by incubating the gels at 4°C for 15–30 min, addition of ice cold PBS and collecting the cell pellet from the fluid after centrifugation. Cells were retrieved from the collagen scaffolds by enzymatic digestion for 45 min at 37°C using collagenase A (Roche). Cell viability was assessed using Annexin V (BD Pharmingen) and 7AAD staining (eBioscience). Flow cytometric analysis was performed on a FACS Calibur (BD Biosciences) or FACS Verse (BD Biosciences) and all data was analyzed using FlowJo software (Version X 10.0 Tree Star).

### Time-lapse microscopy and quantification of cell migration

Migration of individual cells (activated T cells or immature DCs) encapsulated within (RGD-) PIC hydrogels or collagen matrices was monitored by digital time-lapse, bright-field inverse microscopy in a humidified environmental chamber (37°C and 5% CO_2_). Images were collected for 4 h at 2.5 min time intervals with a digital CCD camera (Nikon Diaphot 300 with Hamamatsu C8484-05G CCD Camera, okolab 2D time lapse software). Migration was quantified by tracking 30 randomly selected cells for 3 h with manual tracking FIJI software, beginning >30 min after the start of imaging and using the xy coordinates of cell paths. Cell velocity per cell was calculated as the length of each cell path divided by time, excluding stop phases. The xy trajectories were converted into the mean square displacement (MSD) as previously reported (31). Cells were classified as motile when they show a MSD of >200 um^2^ in the first 2 h of cell tracking. Chemotaxis and Migration Tool software (version 1.01, Ibidi) was used to plot migration trajectories.

### T cell proliferation in 3D matrices

Human pan T cells were stained with CellTrace™ CFSE Cell Proliferation Kit (Invitrogen) to track cell proliferation by flowcytometry. To pre-activate T cells, pan T cells were stimulated overnight with αCD3/αCD28 Dynabeads (Gibco). The following day, T cells were harvested and re-plated into two-dimensional (2D) medium, 3D collagen matrices or 3D RGD-PIC hydrogels at varying cell densities as indicated. Alternatively, unstimulated T cells (1 × 10^6^ cells/ml) were mixed with αCD3/αCD28 Dynabeads (Gibco) or with IL-2 (90-100 IU/mL), PHA (phytohaemagglutinin, 1 μg/mL, Sigma Aldrich) and IL-6 (15 ng/mL, Cell Genix). Subsequently, cells were embedded within the RGD-PIC hydrogels or collagen matrices at varying densities to test *in situ* activation. Cells were recovered from the RGD-PIC gels and collagen gels and proliferation by CFSE dilution and activation were assessed by flowcytometry on a FACS Verse (BD Biosciences). Cell numbers were quantified using a MACSQuant Analyzer 10 (Miltenyi Biotec). Fixable Viability Dye eFluor® 780 (eBioscience) was used to exclude dead cells. Intracellular staining for interferon gamma (IFNy) was done using anti-IFNy (BD Biosciences) and the BD Cytofix/Cytoperm Fixation/Permeabilization.

### *In vivo* PIC gel stability and adoptive transfer of pre-stimulated T cells

Mice were housed at the Central Animal Laboratory (Nijmegen, the Netherlands) where food and water were provided *ad libitum*. This study was carried out in accordance with European legislation. The protocol was approved by the local authorities (CCD, The Hague, the Netherlands) for the care and use of animals with related codes of practice. Animals were randomly allocated to groups. To pre-activate T cells *ex vivo* before adoptive transfer, mouse pan T cells [mouse pan T cell isolation kit (Miltenyi Biotec)] were isolated from the spleens and inguinal lymph nodes of wild-type female C57BL/6J mice (age 5–8 weeks, Charles River) congenic for the CD45.1 marker. T cells were stained with CellTrace™ CFSE and activated for 16 h with immobilized αCD3 monoclonal antibodies (1 ug/mL, clone 17A2, Cell Genix) and αCD28 monoclonal antibodies(2 ug/mL, clone 37.51, Cell Genix). Cell phenotype was determined using flowcytometry staining: CD3 (purity typically >98%), CD69 and CD25 (all Biolegend). Azide-functionalized PIC polymers were dissolved in phenol-red free RPMI medium and were confirmed to be endotoxin free using the LAL test (Lonza). Rheology was performed to confirm adequate gel stiffness. PIC polymers were labeled for 2 h with DCBO-sulfo-Cy5 (Jena Bioscience) and for some experiments with 250 IU/mL IL-2 for 4 h at 4°C. Next, pre-activated CFSE-labeled CD45.1^+^ T cells were mixed with the PIC polymers or with phenol-red free RPMI medium (control), at a final concentration of 1.5 × 10^6^ cells/100 μl. Rheology confirmed that this number of cells did not significantly affect PIC gel stiffness. When IL-2 was attached onto PIC polymers, 250 IU/mL IL-2 was added to the control as well. The PIC polymers were kept on ice and injected s.c. under anesthesia into the dorsal flank of female C57BL/6J mice congenic for the CD45.2 marker (age 5–8 weeks; Charles River, housed at 28°C) in order to discriminate the adoptively transferred T cells from host T cells by flowcytometry. Fluorescent images were collected with the IVIS Lumina imaging system (Cy5 signal: 640 nm, Cy5.5 filter, CFSE signal: 465 nm, GFP filter, Perkin Elmer) after 2 h, 1, 3, 7, 14, 21, or 28 days to investigate gel localization and cellular localization. At designated time points, gels were excised from the dorsal flank and the remaining PIC gel, blood, spleen, draining and non-draining lymph nodes (LNs) were collected. Single cells suspension were made from the excised gels, spleen and LNs by digestion with DNAse (20 μg/mL, Roche) /collagenase type III (1 mg/mL, Worthington) and from the PIC gels by cooling and DNAse/collagenase treatment. The percentage, proliferation and phenotype of CFSE-labeled CD45.1^+^ T cells were quantified by flow cytometry. All flow cytometric analysis was performed on a FACS Verse. A TNF-α ELISA (eBioscience) was performed on serum collected on day 1.

### Immunohistochemistry

On various time points after s.c. injection of Cy5-labeled PIC gels with T cells, PIC gels were resected from the dorsal flank of the mice. The tissues were fixed overnight in 4% PFA at RT. Tissues were embedded in paraffin and 10 um FFPE sections were cut. Antigen retrieval was performed using citrate pH 6.0 (Scytek). Slides were stained with primary anti-CD3 (1:300, clone CD3-12, #MCA 12477, Serotec) and secondary rabbit-anti rat HRP (1:100, Jackson Immunoresearch). Tyramide signal amplicification visualization was performed with the Opal seven-color IHC Kit according to protocol (PerkinElmer) containing fluorophores DAPI and Opal 540. Slides were mounted using Fluormount without DAPI (SouthernBiotech) and scanned using the PerkinElmer Vectra (Vectra 3.0.5; PerkinElmer). Multispectral images were unmixed using spectral libraries using the inForm Advanced Image Analysis software (inForm 2.2.1; PerkinElmer) and analysis was performed by applying an inForm software algorithm (tissue segmentation, cell segmentation, phenotyping tool, and positivity score) based on training with on a selection of 10 representative original multispectral images.

### Statistical analysis

Statistical analyses were performed in GraphPad Prism 5 software using the appropriate testing methods, as indicated in the figure legends. Statistical significance was defined as a two-sided significance level of < 0.05. ns = not significant, ^*^p ≤ 0.05, ^**^p ≤ 0.01, ^***^p ≤ 0.001, ^****^p ≤ 0.0001.

## Results

### Characterization of thermoresponsive polyisocyanopeptide hydrogels

**Figure 1:**
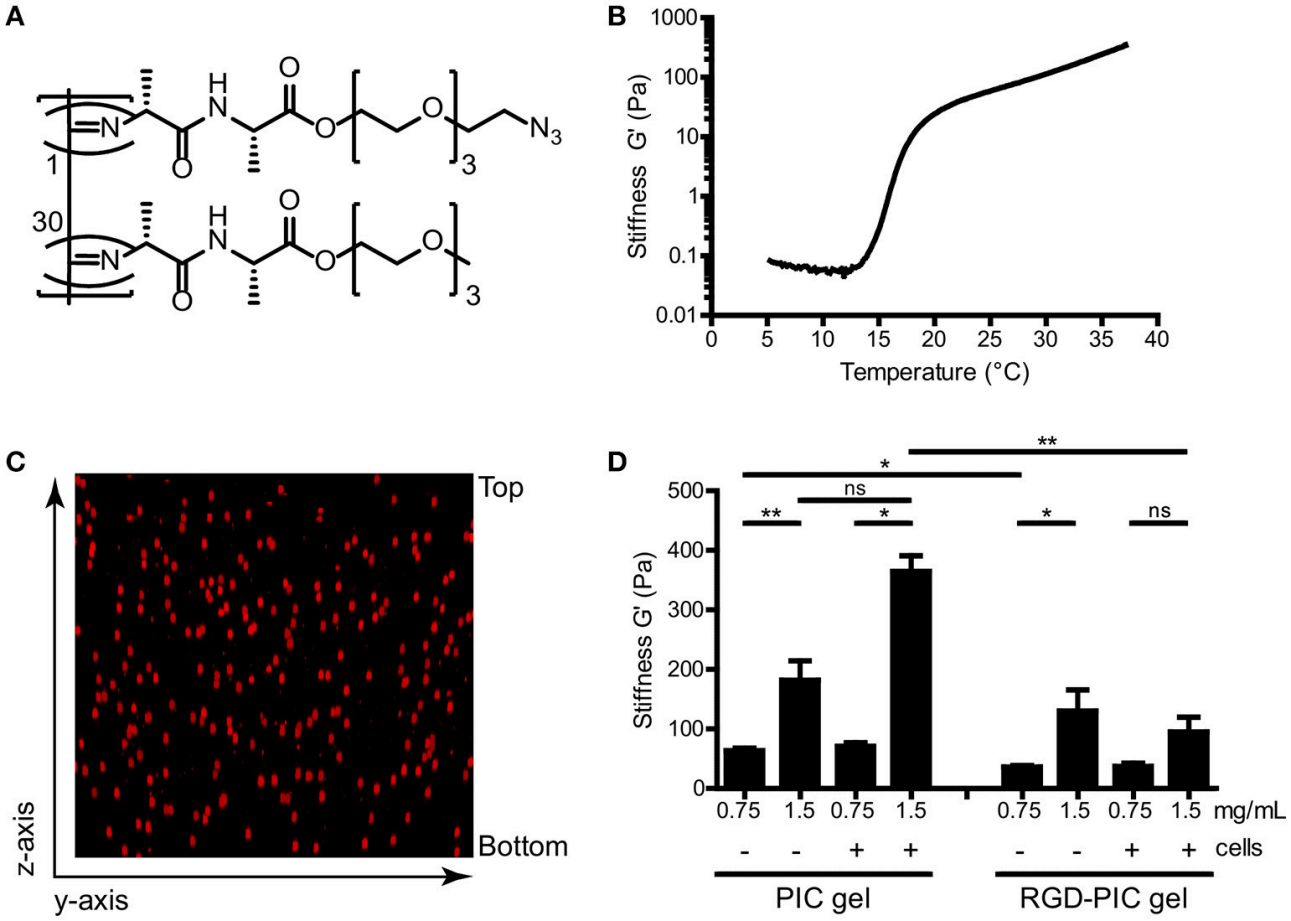
Tri(ethylene glycol)-substituted polyisocyanopeptides (PIC) form soft thermoresponsive gels that can be functionalized via the azide-terminated tail. **(A)** Molecular structure of polyisocyanopeptides substituted with tri(ethylene glycols) and azide functionalization. Azide-functionalized monomer (top) and unfunctionalized monomer (bottom) are co-polymerized in a 1:30 ratio. **(B)** Representative rheological analysis of PIC polymers at 1.5 mg/mL without cells, where G′ as a measure of bulk stiffness is set out against temperature. The material gelates at the LCST of 16°C. **(C)** Z-stack reconstruction of confocal imaging of PKH-26 labeled T cells within PIC hydrogel at 1.5 mg/mL. **(D)** G′ value as a measure of bulk stiffness of PIC hydrogel and RGD-PIC gels, determined with rheological analysis at 37°C. Between 0.5 × 10^6^ and1 × 10^6^ cells/mL were incorporated into the gels. Values represent mean and standard error of the mean (SEM). Data were analyzed with the Kruskal Wallis test and the Dunns post-test. *n* = 3–10 in at least three independent experiments. *p ≤ 0.05, **p ≤ 0.01.

We produced thermoresponsive hydrogels from tri-ethylene glycol-substituted polyisocyanopeptides (PIC). Methoxy-terminated and azide-terminated isocyanide monomers were co-polymerized with a nickel perchlorate (Ni(ClO_4_)_2)_ catalyst in a molar ratio of 1:30 to create azide-containing PIC polymers (Figure 1A). These fully synthetic PIC polymers have an average of one azide functionality every ~3 nm of the polymer chain (28). In order to construct cell-adhesive polymers that would promote interaction of cellular integrin receptors with the matrix, RGD peptide ligands were grafted onto the azide-functionalized PIC polymers via strain-promoted cycloaddition at a ratio of one DBCO-GRGDS per 100 monomers. We analyzed PIC polymers and GRGDS-functionalized PIC polymers (RGD-PIC) by rheology to investigate their bulk stiffness. The polymers demonstrated instantaneous temperature-dependent gelation with an average lower critical solution temperature (LCST) of 16°C (Figure 1B), resulting in the formation of transparent hydrogels at temperatures above the LCST. These thermoresponsive characteristics and gelation temperature are key to warrant straightforward cell encapsulation and injectability of the material.

Next, we mixed primary T cells with cold PIC and RGD-PIC polymer solutions at 4°C and warmed them to 37°C to encapsulate cells during gel formation. As gelation of the PIC polymers occurs virtually instantaneous (within 1 min) upon warming of the polymer solution, cells were encapsulated homogenously in a genuine 3D fashion within the nanoporous network of bundles (Figure 1C). We performed time sweep rheological analysis to measure bulk stiffness of different batches of PIC gel and RGD-PIC polymers, either empty or after encapsulating cells, to investigate the effect of encapsulating adhesion motifs and cells on the material properties. As anticipated, rheology measurements indicated that PIC and RGD-PIC hydrogels exhibit a concentration-dependent stiffness and form soft gels with a stiffness ranging between 0.03 and 0.4 kPa (Figure 1D). The presence of RGD within the hydrogels reduced the average bulk stiffness of the gels, especially for PIC gels with higher stiffness. The incorporation of cells did not affect stiffness significantly at a density of 0.5–1 × 10^6^ cells/mL of gel.

### Polyisocyanopeptide hydrogels are biocompatible and sequester T cells

**Figure 2:**
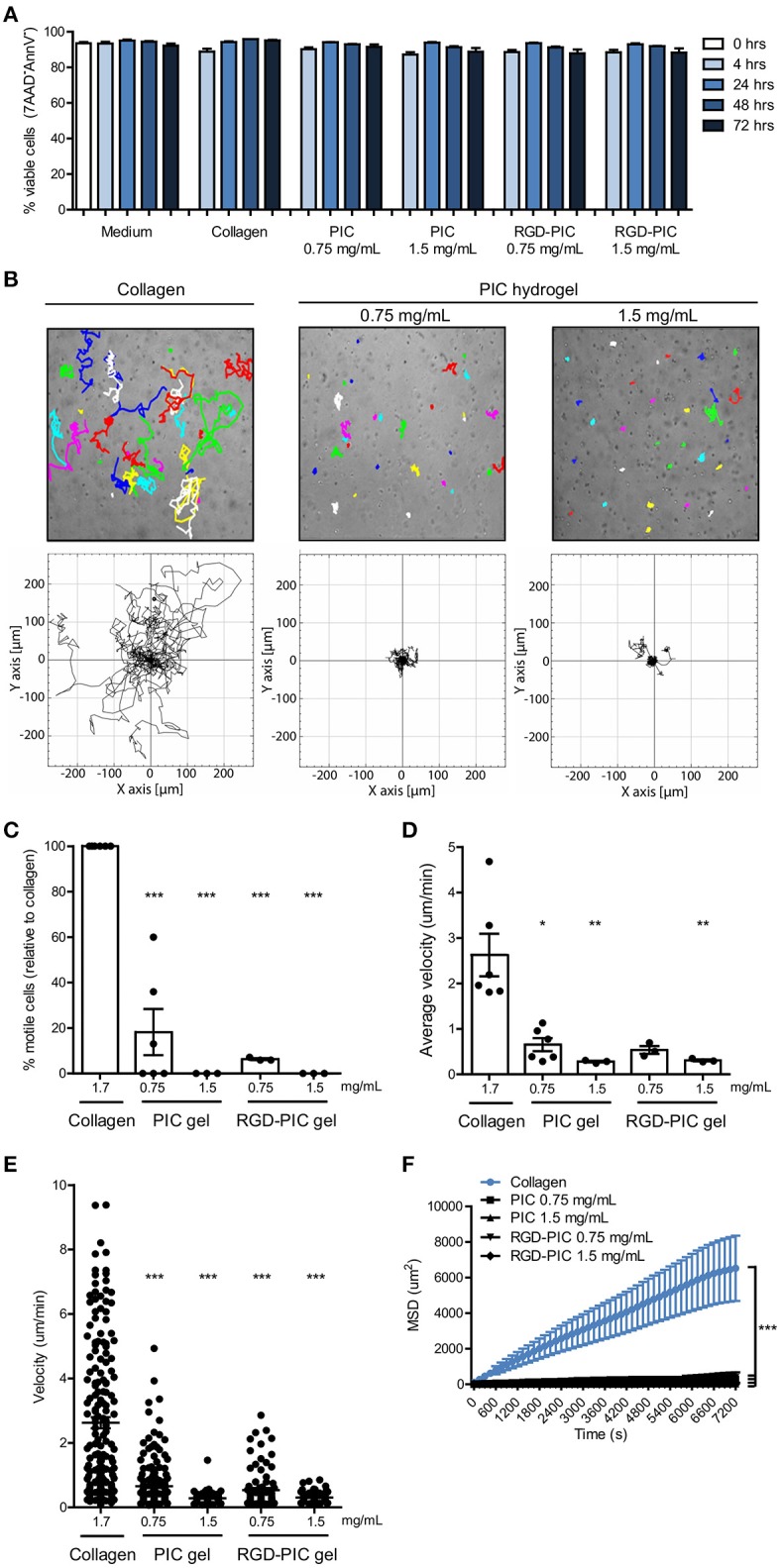
PIC hydrogels support T cell survival and restrict T cell migration within the matrix. **(A)** Viability of αCD3/αCD28 dynabead-activated T cells in medium, collagen gels, and (RGD-) PIC hydrogels, defined as the percentage of 7AAD^−^Annexin V^−^ cells. *n* = 3 in one experiment. **(B)** Top: Representative images of 3 h cell tracks of individual activated T cells in collagen gels and PIC hydrogels. Each dot and/or track represents one randomly chosen cell out of 30. Bottom: Trajectories of 30 individual activated T cells from one representative experiment. **(C)** The percentage of motile cells relative to the number of motile cells in collagen. Cells are classified as motile when they show a mean squared displacement (MSD) of >200 um^2^ in the first 2 h of cell tracking. **(D,E)** The average velocity depicted for the average of all cells **(D)** or per single cell **(E)** based on tracking of 30 randomly chosen cells for 3 h in collagen gels and (RGD-) PIC hydrogels. **(F)** The mean squared displacement of 30 cell tracks in collagen and (RGD-) PIC hydrogels that were tracked for 2 h. **(A,C–F)** Values represent mean and SEM. **(C–E)** Data were analyzed with the Kruskal Wallis test and the Dunns post-test. **(F)** A two-way ANOVA with a Bonferroni post-test was performed to test for statistical significance. **(C–F)** *n* = 6 for collagen and PIC gel 0.75 mg/mL and *n* = 3 for others in at least three independent experiments. *p ≤ 0.05, **p ≤ 0.01, ***p ≤ 0.001.

To establish whether PIC and RGD-PIC hydrogels are biocompatible and can promote the survival of primary T cells, we embedded pre-stimulated primary human T cells within (RGD-) PIC gels and cultured them at 37°C. Due to the thermoreversible behavior of the hydrogels, cells could be easily retrieved from the 3D matrices for phenotypical analysis by incubating the gels at 4°C. Primary T cells remained highly viable within PIC and RGD-PIC hydrogels for at least 3 days at levels comparable to that in medium and collagen type I hydrogels (Figure 2A). The wide applicability of PIC hydrogels is further supported by the notion that primary human dendritic cells (DCs) and primary natural killer (NK) cells are also viable within the PIC hydrogels at a similar level as cells cultured in medium or collagen (Supplementary Figures 1A,B), demonstrating that PIC hydrogels support 3D cell culture of a wide variety of primary immune cells.

The 3D matrix that is formed by the bundles of the PIC polymers consist of a tight nanoporous network with an estimated average pore size of 200 nm, which is physically crosslinked by bundling of polymer chains at temperatures above the LCST (27). We aimed to characterize the migratory behavior of immune cells within PIC hydrogels to understand how immune cells behave in this 3D culture system. We tracked non-directional migration of pre-activated T cells in PIC and RGD-PIC hydrogels within 4 h after their encapsulation, and compared it to random migration in collagen type I hydrogels with pore diameters of 2–6 um as a mimic of a natural 3D matrix that supports immune cell migration (32). Time-lapse microscopy revealed that primary T cells hardly migrate through PIC hydrogels, in contrary to T cells encapsulated in collagen gels (Figures 2B–F). The ability to migrate was directly dependent on gel concentration as increasing PIC gel concentration to 1.5 mg/mL completely arrested cell migration, possibly as a result of enhanced gel stiffness (Figure 1D). A minor decrease in pore size could contribute to this as well as pore size correlates inversely with polymer concentration (27). Next, we investigated whether functionalizing PIC gels with RGD integrin-binding motifs would promote T cell migration. RGD peptide ligands interact with α4β1 integrins on T cells (33) and are frequently incorporated into synthetic biomaterials in order to promote cellular adhesion and enhance interaction of cells with biomaterials (34). T cells did not display increased migration through gels bearing RGD adhesion-motifs compared to non-functionalized gels, even though RGD-PIC hydrogels generally have a lower bulk stiffness than PIC gels (Figures 2B–F). This could suggest that the compact bundled network of the PIC hydrogels is the main determining factor of cellular migration. Alternatively, it could also be a result predominantly integrin-independent amoeboid migratory behavior that T cells utilize (35), which is therefore not enhanced by the presence of integrin-binding motifs. Tracking random migration of immature myeloid DCs demonstrated that they displayed migratory behavior similar to that of T lymphocytes within PIC gels, as they were restricted in their migration through the tight network with small pores (Supplementary Figures 1C–F). However, DCs retained higher levels of migration compared to T cells in 0.75 mg/mL PIC hydrogels, probably because of differences in their ability to deform their nucleus as this is one of the determining factors when physical restrictions are imposed on migration (32). The finding that DCs preserve some of their migratory capacity within the PIC matrix implies that the polymer network does not inhibit the intrinsic migratory function of cells but instead poses a physical barrier. These observations together with the notion that immune cells are viable within (RGD-) PIC gels (Figure 2A, Supplementary Figures 1A,B) suggest that cell migration is restricted because of physical restraints imposed by the PIC polymer bundles which sequesters cells within the 3D matrix.

Next, we studied whether the absence of immune cell migration in PIC gels affects their inherent capacity to become activated by stimulatory cues. Activation and proliferation of primary T cells embedded in RGD-PIC hydrogels could be induced by introducing T cell-activating αCD3/αCD28 dynabeads, but only when relatively high numbers of cells and high numbers of beads were mixed together which statistically enhances the chance of a T cell-activating bead and a non-migratory T cell to interact (Supplementary Figures 2A–C). This shows that sequestered T cells still have the potential to become activated and expand. Moreover, these T cells can also be activated using soluble T cell activators such as PHA/IL-6/IL-2 (Supplementary Figure 2D), further confirming that the inherent capacity of encapsulated cells for activation and proliferation is not affected by the dense RGD-PIC gel matrix.

Collectively, these data show that PIC and RGD-PIC hydrogels are biocompatible matrices that can be used to culture primary T cells and other primary immune cells in a 3D environment. The dense polymer network physically restricts immune cell migration but does not affect T cell ability to become activated or expand.

### Primary T cells can be expanded at high cell densities within PIC hydrogels

**Figure 3:**
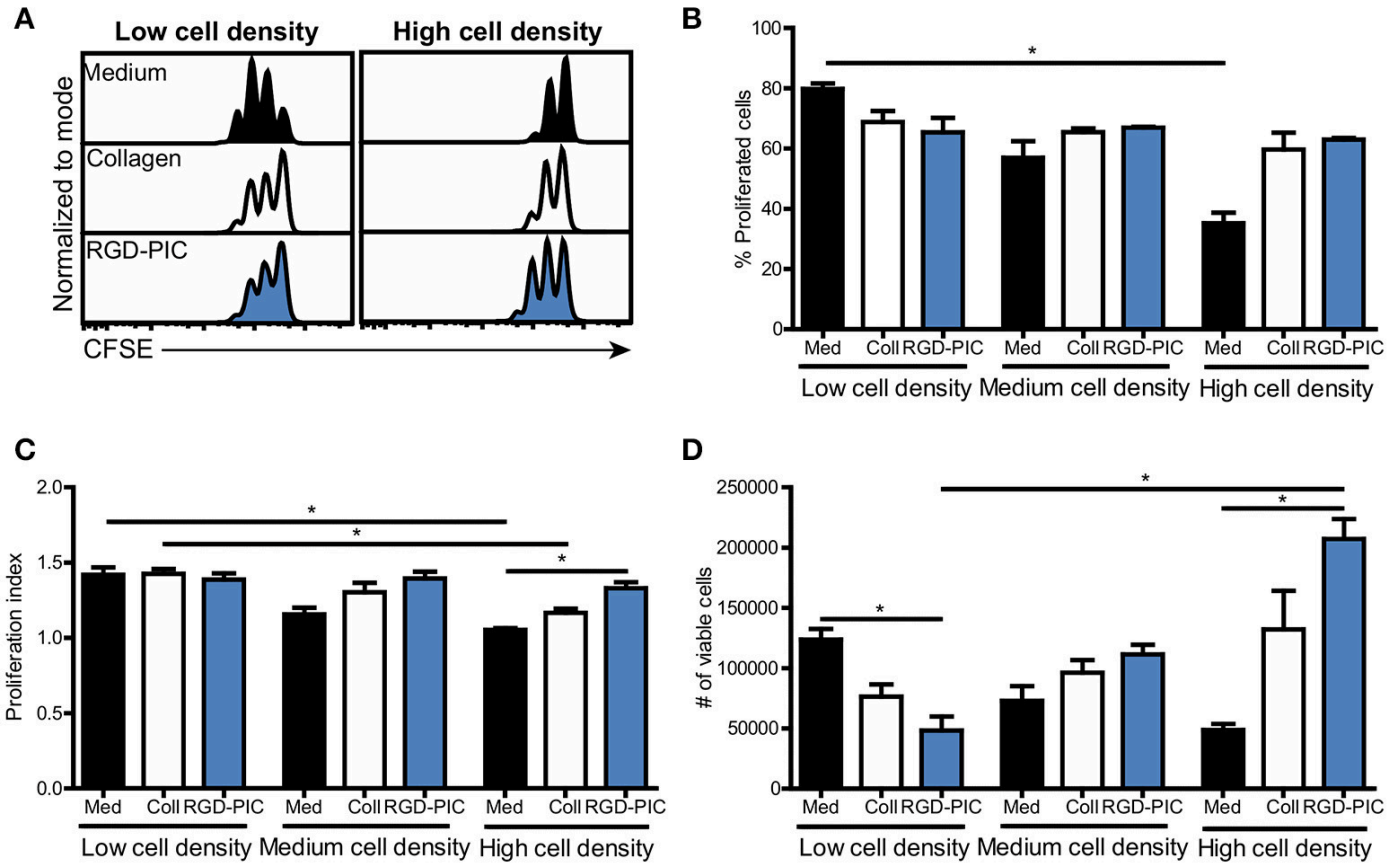
Pre-stimulated T cells display extensive proliferation within RGD-PIC hydrogels, even at high cell densities. **(A)** Representative histograms of CFSE-labeled pre-activated pan T cells cultured in medium, collagen gels or RGD-PIC gels (1.5 mg/mL) for 3 days. Low cell density: 1 × 10^6^ cells/mL, medium cell density: 2.5 × 10^6^ cells/mL, high cell density: 5 × 10^6^ cells/mL. **(B–D)** The percentage of proliferated T cells defined as cells that have undergone at least 1 proliferation cycle **(B)**, the proliferation index defined as the number of cycles of proliferating T cells **(C)** and a quantification of the absolute number of cell retrieved **(D)** 72 h after transferring pre-activated T cells to medium, collagen or RGD-PIC gels at varying cell densities. Low cell density: 1 × 10^6^ cells/mL, medium cell density: 2.5 × 10^6^ cells/mL, high cell density: 5 × 10^6^ cells/mL. **(B–D)** Values represent mean and SEM. Data were analyzed with the Kruskal Wallis test and the Dunns post-test. *n* = 2 or 3 in at least two independent experiments. *p ≤ 0.05.

In adoptive T cell therapy approaches such as TIL therapy, T cells need to be expanded to high numbers of tumor-reactive T cells as typically 10^10^ cells are administered to patients (36). Therefore, we tested if PIC hydrogels could facilitate and promote the proliferation of pre-activated T cells embedded within the 3D scaffold. We incorporated human pan T cells into PIC hydrogels functionalized with RGD to support integrin-mediated T cell interaction with the matrix. Non-activated T cells cultured with RGD-PIC hydrogels did not proliferate, demonstrating that RGD-PIC scaffolds do not intrinsically induce T cell expansion (Supplementary Figure 3A). Next, we activated T cells with αCD3/αCD28-coated dynabeads (Supplementary Figure 3B) and transferred these pre-activated T cells into 2D medium, collagen gels or RGD-PIC hydrogels at a density of 1 × 10^6^ cells/mL and studied their proliferation. Pre-activated lymphocytes expanded rapidly and extensively in RGD-PIC gels similar to that of T lymphocytes embedded in collagen gels or in 2D medium, with proliferation rates ranging from 60 to 80% (Figures 3A–C, low cell density). We hypothesized that the 3D environment provided by the RGD-PIC gels could be beneficial for expansion of T cells at high quantities and therefore we increased the density of T cells within the gels with 2.5 times (“medium cell density”) and 5 times (“high cell density”). Notably, T lymphocytes propagated extensively in RGD-PIC gels and collagen gels at high cell densities, in contrast to T cells cultured in 2D in medium where cellular crowding inhibited T cell expansion (Figures 3A–C). Quantification of the number of T cells demonstrated that significantly more T cells could be retrieved from RGD-PIC gels compared to T cells cultured in 2D medium when they were cultured at high cell densities (Figure 3D). On the contrary, at low cell densities most cells were retreived from 2D medium cultures, eventhough T cell proliferated at similar speed in collagen and RDG-PIC gels. This indicates that cells are lost during cell recovery, which might be caused by collagenase treatment required to retrieve cells from collagen. For RGD-PIC gels, this is probably the result of a higher viscosity compared to medium, indicating that optimizaton of the cell retrieval process could further increase the number of T cells that can be obtained. The percentage of IFNy-producing T cells in RGD-PIC gels was comparable to that of T cells expanded in collagen and medium (Supplementary Figure 3C), again suggesting that RGD-PIC gels do not inhibit T cell functions including cytokine production. These data confirm that the RGD-PIC hydrogels are able to support the propagation of T cells and can promote the rapid proliferation of pre-stimulated T cells at high cell densities.

### Polyisocyanopeptide polymers form stable gels *in vivo* and are non-immunogenic

After having established that PIC gels support the survival and proliferation of T cells as 3D culture systems *in vitro*, we wanted to explore the feasibility of applying PIC gels *in vivo*. One of the key advantages of the thermoresponsive properties of PIC hydrogels is the opportunity to introduce these into the body in a minimally invasive manner via *in vivo* gelation after needle-mediated injection of a cold PIC polymer solution, as opposed to pre-formed scaffolds that require implantation. We set out to explore the potential of PIC hydrogels for the subcutaneous (s.c.) delivery of immune cells via injection in order to deliver high numbers of cells concentrated in a relatively small volume into the tissue, whilst retaining the effector functions of the expanded T cells (Figure 3C, Supplementary Figure 3C). The PIC gel could in this way be used to dictate cellular localization and at the same time provide a supportive matrix that promotes cellular proliferation.

**Figure 4:**
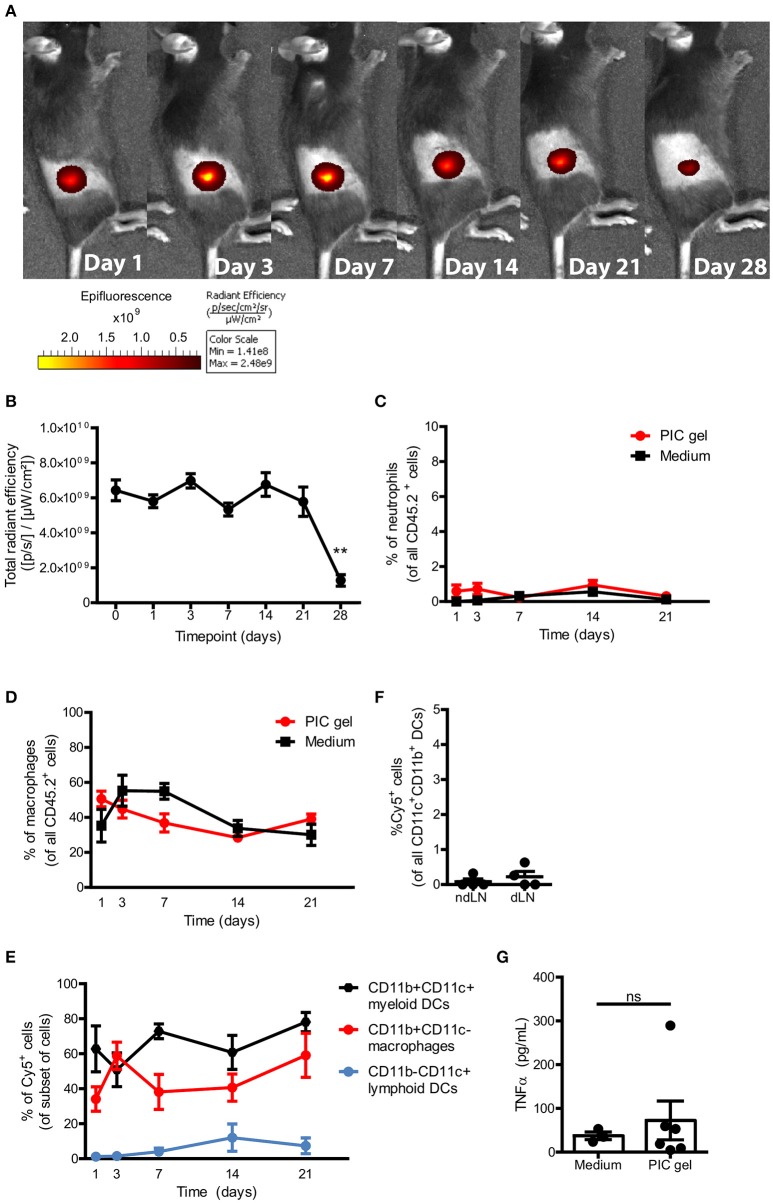
PIC gels form stable gels *in vivo* that do not cause inflammation. **(A,B)** Representative example **(A)** and quantification **(B)** of the radiant efficiency of IVIS imaging of mice s.c. injected with 100 μl Cy5-labeled 1.5 mg/mL PIC polymers mixed with T cells in the dorsal flank. *N* = 6–20 in three independent experiments. **(C,D)** The percentage of SSC^high^Ly6G^+^ neutrophils **(D)** and CD11b^+^CD11c^−^ macrophages **(E)** of all alive CD45.2^+^ cells that where surrounding or inside the PIC gel, compared to a similar region of skin in mice injected with medium and T cells. *N* = 7–9 in at least 3 independent experiments. **(E)** The percentage of Cy5^+^ cells of the respective subset surrounding or inside the PIC gel of mice injected with Cy5-labeled PIC gel. *n* = 7–9 in at least 3 independent experiments. **(F)** The percentage of Cy5^+^ cells of all CD11c^+^CD11b^+^ DCs in the non-draining lymph node (ndLN) and draining lymph node (dLN) on day 7. *n* = 4 in 1 independent experiment. **(G)** The level of TNFα in serum 1 day after injecting mice with Cy5-labeled PIC polymers or medium mixed with T cells. **(B–G)** Values represent mean and SEM. **(B)** Data were analyzed with the Kruskal Wallis test and the Dunns post-test. **p ≤ 0.01 **(C–E)** Data were analyzed with the a two-way ANOVA and Bonferroni post-test. **(G)** Data were analyzed with the Mann Whitney test. ns, not significant.

To investigate the gelation of cold PIC polymers *in vivo* after subcutaneous injection, we labeled azide-functionalized PIC polymers with DBCO-sulfo-Cy5. We injected 100 μl of cold 1.5 mg/mL PIC polymers mixed with pan T cells suspended in medium s.c. into the dorsal flank of C57Bl/6J mice. Fluorescent imaging starting 2 h after injection (day 0) revealed that a confined structure was formed (Figure 4A), suggesting that gelation *in vivo* occurs rapidly before polymers get dispersed over the subcutaneous space. The PIC gel remained localized in the dorsal flank for at least 4 weeks and significantly decreased in fluorescence intensity after 4 weeks (Figures 4A,B), which could suggest that the gel is degrading over time and washed away from the injection site when gel stability reaches a lower limit. Importantly, mice did not show any signs of distress nor weight loss during the entire 4 weeks after gel administration, indicating that PIC gels are well tolerated. To study whether the PIC gel induces inflammation, we resected the PIC gels and the surrounding tissue to compare the presence of neutrophils and macrophages to that of a similar sized piece of skin close to the injection site of control mice injected with T cells in medium without PIC polymers. PIC gels did not induce any recruitment or influx of inflammatory neutrophils or macrophages toward or into the PIC gels compared to control mice (Figures 4C,D), indicating that the scaffolds are non-immunogenic. This was confirmed by the observation that predominantly CD11b^+^CD11c^−^ macrophages and CD11b^+^CD11c^+^ myeloid DCs take up some of the Cy5-labeled PIC polymers (Figure 4E) but these Cy5^+^ DCs do not migrate toward the draining lymph nodes of these mice (Figure 4F), suggesting that DCs do not receive any activation cues as a result of polymer uptake. Moreover, we did not detect any differences in the serum levels of TNFα 1 day after injection between mice injected with PIC gels vs. mice injected with medium (Figure 4G). Thus, s.c. injection of PIC polymers together with T lymphocytes results in the formation of a stable PIC gel without induction of local or systemic immune activation. This suggests that PIC hydrogels are non-immunogenic, biocompatible and can be safely used for *in vivo* immunomodulation.

### Pre-activated T cells delivered via PIC gels maintain their function and are slowly released into the environment

**Figure 5:**
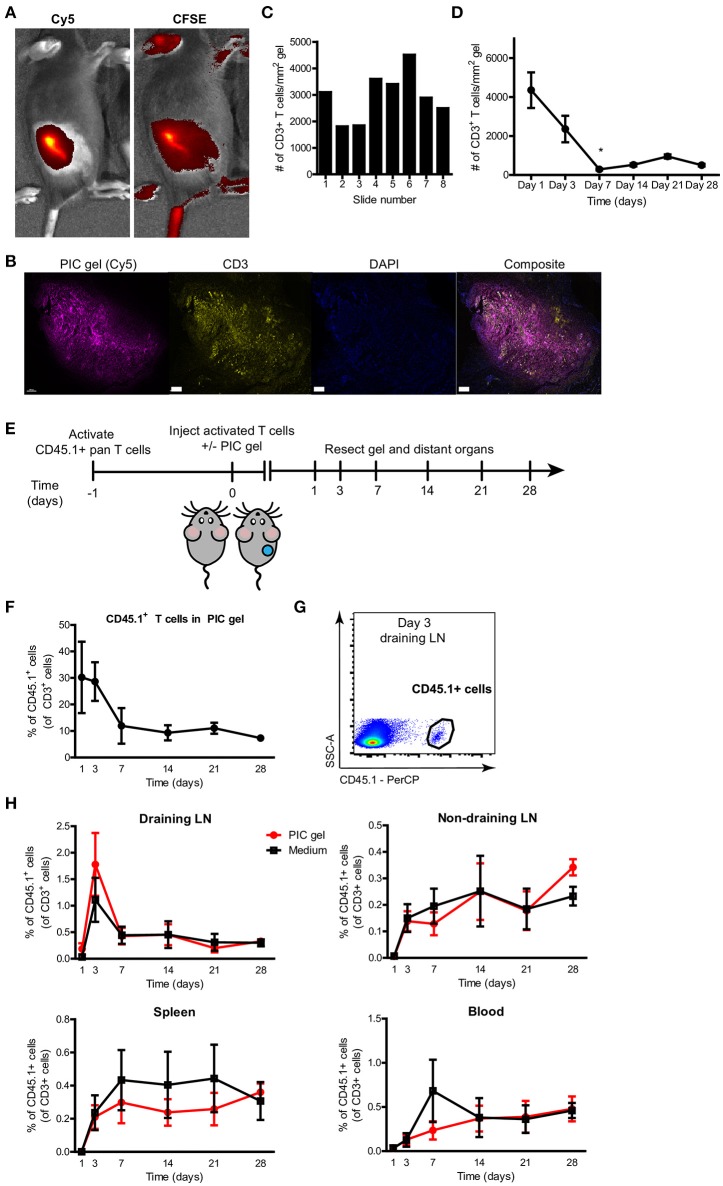
T cells are encapsulated within PIC gels after *in vivo* gelation and are released into the blood and distant organs. **(A)** Representative IVIS fluorescent images of the radiant efficiency of a mouse injected s.c. with 100 ul Cy5-labeled 1.5 mg/mL PIC gel mixed with CFSE-labeled pan T cells in the dorsal flank. **(B)** Representative image of a 10 μm section of FFPE-treated PIC gel in the skin 1 day after injection. Scale bar = 400 um. **(C)** Representative quantification of the number of CD3^+^ T cells per mm^2^ PIC gel of 10 um sections through the PIC gel with typically 20–30 um in between each section (1 day after injection). **(D)** Quantification of the average number of CD3^+^ T cells per mm^2^ PIC gel of 10 um sections through the PIC gel (*n* = 2 or 3 per timepoint in 2 independent experiments). Data analyzed with Kruskal Wallis test with dunn's post test relative to day 1, *p ≤ 0.05. **(E)** Setup of experiment to study release of T cells from PIC gels after injection. **(F)** Quantification of the percentage of CD45.1^+^ cells of all CD3^+^ T cells retrieved from PIC gels and surrounding skin. *n* = 5–7 in at least 3 independent experiments for timepoints day 1–21, *n* = 2 in 1 independent experiment for day 28. Data analyzed with Kruskal Wallis test with dunn's post test relative to day 1, not significant. **(G)** Representative flow cytometry plot demonstrating CD45.1^+^ cells gated on CD3 expression in the draining lymph node (LN) on day 3 after injection of PIC gel with CD45.1^+^ T cells. **(H)** Quantification of the percentage of CD45.1^+^ cells of all CD3^+^ T cells in the draining lymph node (LN), non-draining lymph node, spleen and blood. *n* = 7–9 in at least three independent experiments for timepoints day 1–21, *n* = 6 in one independent experiment for timepoint day 28. **(D,F,H)** Values represent mean and SEM.

Next, we investigated whether T cells could be encapsulated within PIC gels after s.c. injection and *in vivo* gelation. We mixed 1.5 × 10^6^ CFSE-labeled pre-stimulated primary mouse T cells per 100 μl of cold Cy5-labeled PIC polymers. After s.c. injection, we could see a clear colocalization of the Cy5 signal coming from the PIC gel together with the CFSE signal from the T lymphocytes (Figure 5A). We resected the PIC gels on various time points after injection and performed immunohistochemistry for CD3 to localize and quantify the number of CD3^+^ T cells within the gel. T cells could be identified as high-density clusters within the polymers of the PIC hydrogel and localized mainly within or in close proximity to the gel (Figure 5B), confirming that T cells are encapsulated within PIC gels after *in vivo* gelation. This implies that T cells can tightly interact with the PIC polymers after co-delivery. Multispectral image analysis through different sections at varying heights of the PIC gel revealed that the scaffold contains a relatively consistent number of T cells throughout the entire gel construct (Figure 5C). Immunohistochemistry indicated that 1 day after injection there was an average of more than 4000 CD3^+^ T cells per mm^2^ PIC gel. Over time, the number of T cells within PIC gels gradually diminished (Figure 5D).

In order to use PIC gels as cellular delivery vehicles of T lymphocytes for applications such as ACT, it is crucial that pre-activated T cells retain their proliferative capacity and functionality after administration while preserving their ability to move out of the scaffold into the environment. In particular, we were interested in the release kinetics of T lymphocytes co-delivered with PIC polymers, as we observed that PIC gels restrict T cell migration *in vitro*. To this end, we mixed pre-activated mouse T cells (CD45.1^+^) with cold PIC polymer and adoptively transferred these by s.c. injection in the dorsal flank of CD45.2^+^ mice, and compared it to injection of T cells without PIC gel (Figure 5E). We studied T cell migration away from the injection site by investigating the presence of CD45.1^+^ cells in the spleen, draining lymph node (dLN), non-draining lymph node (ndLN) and blood at various time points. The relative amount of CD45.1^+^ T cells in the PIC gel determined by flowcytometry decreased starting from day 1 after injection until day 28 (Figure 5F), confirming data obtained by CD3^+^ staining of PIC gel sections (Figure 5D). A decrease of T cells within PIC gels was accompanied by an accumulation of CD45.1^+^ T cells in the dLN at day 3 after injection (Figure 5G). Subsequently, we detected a steady increase of adoptively transferred T cells in the non-draining LN, spleen and blood of the recipient mice up to 4 weeks after injection (Figure 5H). Strikingly, the release kinetics for T cells mixed with PIC polymers vs. T cells alone was similar. This suggests that T cells can readily migrate out of the PIC gels even though they are clustered within the scaffold 1 day after injection (Figure 5B), and they demonstrated a significantly restricted cellular migration *in vitro* on the short term (Figure 2). The release of T cells from PIC gels *in vivo* is likely a result of a decrease in gel stability and finally degradation over time. Thus, PIC gels incorporate T cells after *in vivo* gelation and allow egress of T lymphocytes from the injection site *in vivo*.

Finally, we performed a detailed characterization of the phenotype and function of adoptively transferred CD45.1^+^ T cells alone or in the context of PIC gels. The PIC gels did not affect the proliferative capacity (Supplementary Figure 4A) or the effector/memory phenotype balance of transferred T lymphocytes (Supplementary Figures 4B,C). Moreover, PD-1 expression on these cells was not changed compared to T cells injected without PIC gel (Supplementary Figure 4D). Together, these results suggest that the PIC gel does not negatively impact the quantity, release kinetics or quality of transferred T lymphocytes after s.c. delivery.

## Discussion

The use of biomaterial-based scaffolds as 3D culture systems and cellular delivery vehicles is a promising approach to improve the efficacy of immunotherapy for cancer and reduce toxicity. Scaffold properties need to be selected and tested systematically to match these to future applications. Here, we characterize the potential of PIC hydrogels as 3D culture systems for primary T cells. We report that PIC gels support expansion of pre-stimulated T lymphocytes at high cell densities without affecting cell functionality, in contrast to less physiologically relevant 2D culture systems where crowding may hamper cellular proliferation (37). This demonstrates that PIC hydrogels are attractive 3D scaffolds to propagate pre-stimulated T cells to benefit T cell expansion protocols for ACT, as it can support extensive T cell expansion in a small volume while preserving T cell function. As such, we hypothesize that PIC hydrogels can be used to enhance efficiency of current T cell expansion protocols and that they could reduce the length of the *in vitro* culture period that precedes re-administration of tumor-reactive T cells, which positively affects the quality of the T cell infusion product (38, 39). The thermoreversible behavior of PIC hydrogels facilitates straight forward cell encapsulation into the 3D matrix and importantly, allows for rapid retrieval of cells. This is a great benefit over many other 3D culture systems where typically mechanical or enzymatic disruption is required to retrieve cells, which can affect cell survival, cell surface markers, phenotype or gene expression (40).

The mechanical properties of scaffolds are of high importance for stability during cell culture and may affect cellular behavior. We establish that the dense bundled network of PIC gels restricts primary T cell and DC migration, which is not mitigated by incorporating integrin-binding RGD motifs. This is likely caused by the physical restraints imposed on encapsulated cells by the tight bundled network with 200 nm pores (27). T cell sequestration does not affect their ability to become activated or proliferate, but has implications on how PIC gels can be applied as cells cannot freely interact with immobilized activating cues incorporated into the system. To fully apprehend the potential of PIC gels as 3D cell culture systems for various cell types, the precise relationship between gel stiffness, polymer concentration, the presence of adhesive ligands and migration propensity needs to be established and specified per cell subtype. It is important to take into account how gel stability and stiffness change over time, as prolonged use of PIC gels in 3D cell culture will decrease gel stiffness and permit cellular migration (41).

We exploit the thermosensitive behavior of PIC polymers to trigger gelation into 3D matrixes. This is highly advantageous as gelation occurs under physiological circumstances and does not require any crosslinkers, organic solvents or potentially toxic agents to induce gel formation (42, 43). The tri-ethylene glycol-substituted PIC polymers used in this study have a LCST of 16°C, which ensures stable gels at physiological temperatures of 37°C as gels become more stiff at higher temperatures (27). A consequence of this LCST is that polymer solutions need to be kept cool below 16°C during the handling time and prior to injection. The LCST of these PIC polymers can be tuned for instance by the addition of salts (44), but this requires careful optimization as changing the LCST will also affect gel stiffness and stability at 37°C. Thermally-induced gelation permits minimally invasive delivery *in vivo* via needle-mediated injection which precludes the need for a surgical procedure for scaffold implantation. Injected cold PIC polymers are well tolerated, form stable gels after *in vivo* gelation and notably do not induce a local or systemic inflammatory response, suggesting that these polymers can be safely used *in vivo*. In particular, we observe no neutrophil or macrophage recruitment toward the gel, which is an important indicator for a lack of scaffold immunogenicity and a crucial factor for biocompatibility (45–48). The mode of delivery and precise scaffold formulation is central in this context, as implantation of RGD-PIC gels within silicon molds has previously been found to induce mild granulocyte recruitment (41). This results from a tissue damage response following implantation together with immune activation due to RGD peptide ligands immobilized onto the polymers (49, 50). Moreover, we observe that local *in vivo* gel fluorescence diminishes after 4 weeks, which implies PIC gel degradation and is favorable with respect to biocompatibility. Degradation is presumably a result of disruption of the non-covalent interactions that hold the PIC polymer bundles together, until the hydrogel is too weak to stay intact and is cleared from the subcutaneous space. Thus, our findings demonstrate that PIC polymers can be safely used *in vivo* and can efficiently be delivered via injection.

By mixing pre-activated T cells with PIC polymers, we could locally deliver high numbers of T cells *in vivo* within PIC gels. T cells move toward distant organs from PIC gels at a speed similar to that of T cells injected without scaffold, even though we observed restricted T cell migration *in vitro*. This can be explained by distinct gelation behavior after *in vitro* vs. *in vivo* gel formation (51, 52), as tissue pressure and fluid drainage may affect polymer gelation *in vivo*. Another factor is probably weakening of PIC gels over time, causing T cells to egress out of the scaffolds (41). T cells circulate systemically and move into distant organs, while preserving their proliferative capacity and functionality. This is critical to ensure that PIC-delivered T cells can migrate toward target sites and execute their (effector) functions. We demonstrate that cells are in close proximity of PIC polymers after injection and are migratory, suggesting that extensive cell-matrix interaction is possible. This provides the opportunity to exploit the azide handles present on PIC polymers using bio-orthogonal click chemistry to co-deliver a wide variety of biomolecules that can steer T cell survival, phenotype or function after administration. Covalent attachment of T cell survival factors or activating cues onto the polymers promotes sustained availability of these factors at a localized area, in contrast to rapid diffusion of biomolecules administered in a soluble fashion. We hypothesize that the introduction of T cell-stimulating cues in the PIC hydrogels may contribute to promoting T cell viability and functionality in order to outperform T cell delivery through bolus injection. This can be exploited by introducing IL-2 or IL-15 agonists into the PIC gels that may locally enhance T cell viability and persistence (18, 53), while circumventing the toxicity associated with high dose bolus IL-2 (24). Alternatively, scaffolds bearing T cell-activating cues such as agonistic CD3 and CD28 antibodies (54) alone or together with αCD137 and IL-15 agonists (55) can be used to promote vigorous *in situ* T cell expansion and improve functionality. Anti-tumor immune responses can be further boosted by presenting stimulator of interferon genes (STING) agonists (56). These strategies are promising to increase efficacy of ACT by locally stimulating and expanding adoptively transferred T lymphocytes in a tunable 3D environment compared to conventional bolus injection of pre-stimulated T cells (55, 56). In addition, this may reduce the need to support T cell engraftment using toxic co-treatments such as lymphodepleting chemotherapy and IL-2 (18).

As we describe that PIC hydrogels are not only suitable for cell culture of primary T cells but also support DC survival to a similar extent as medium, we speculate that PIC gels may be suitable for the localized delivery and local stimulation of DCs for DC-based cancer vaccination approaches. In this setting DC-stimulating cues such as covalently attached TLR ligands and tumor antigens can be grafted onto the polymers to create an immunostimulatory niche (10, 57, 58), although careful consideration with respect to the local persistence of TLR ligands and tumor antigens is pivotal to induce anti-cancer immunity rather than tolerance (59).

Our findings build on previous work that reported that PIC gels are biocompatible and can support the culture of various cell types including mesenchymal stem cells, adipocytes, melanoma cells, fibroblasts and endothelial cells (28, 41). This highlights the biocompatible nature of PIC hydrogels and puts them forward as versatile scaffolds that are immunologically silent. Their synthetic nature and azide-handles create a flexible and controlled platform that is readily applied for immunomodulation, both *in vitro* and *in vivo*. As such, PIC hydrogels are highly valuable tools as 3D cell culture systems and cellular delivery vehicles, for which the choice and formulation need to be tailored to the desired application.

## Author contributions

JW, YD, and LE performed the experiments. DV, RD, and RH prepared and characterized the polymers. AvD provided technical assistance and performed immunohistochemistry experiments together with JW. JW, YD, RH, and LE designed experiments and interpreted the data. AR, JT, and CF supervised the study. JW and CF wrote the manuscript with input from all authors.

### Conflict of interest statement

The authors declare that the research was conducted in the absence of any commercial or financial relationships that could be construed as a potential conflict of interest.
