# Supplementary material for: Injectable Biomimetic Hydrogels as Tools for Efficient T Cell Expansion and Delivery
Source: Front Immunol. 2018 Nov 28;9:2798. doi: 10.3389/fimmu.2018.02798 (PMC6279891; doi:10.3389/fimmu.2018.02798)
Supplement: Supplementary file 1 [file Presentation_1.pptx]

## Slide 1
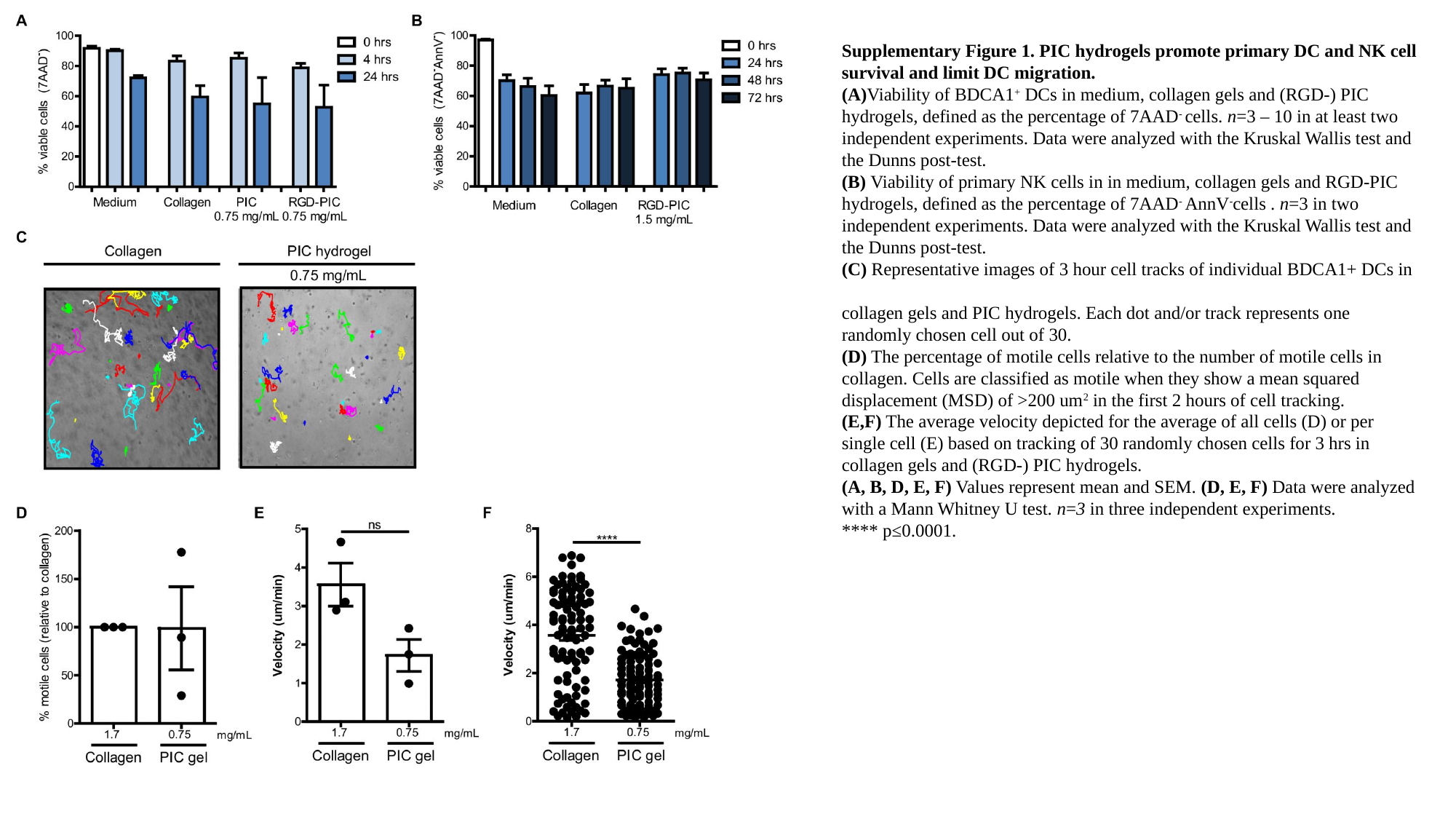

Supplementary Figure 1. PIC hydrogels promote primary DC and NK cell survival and limit DC migration.
(A)Viability of BDCA1+ DCs in medium, collagen gels and (RGD-) PIC
hydrogels, defined as the percentage of 7AAD- cells. n=3 – 10 in at least two
independent experiments. Data were analyzed with the Kruskal Wallis test and
the Dunns post-test.
(B) Viability of primary NK cells in in medium, collagen gels and RGD-PIC
hydrogels, defined as the percentage of 7AAD- AnnV-cells . n=3 in two
independent experiments. Data were analyzed with the Kruskal Wallis test and
the Dunns post-test.
(C) Representative images of 3 hour cell tracks of individual BDCA1+ DCs in
collagen gels and PIC hydrogels. Each dot and/or track represents one
randomly chosen cell out of 30.
(D) The percentage of motile cells relative to the number of motile cells in
collagen. Cells are classified as motile when they show a mean squared
displacement (MSD) of >200 um2 in the first 2 hours of cell tracking.
(E,F) The average velocity depicted for the average of all cells (D) or per
single cell (E) based on tracking of 30 randomly chosen cells for 3 hrs in collagen gels and (RGD-) PIC hydrogels.
(A, B, D, E, F) Values represent mean and SEM. (D, E, F) Data were analyzed
with a Mann Whitney U test. n=3 in three independent experiments.
**** p≤0.0001.

## Slide 2
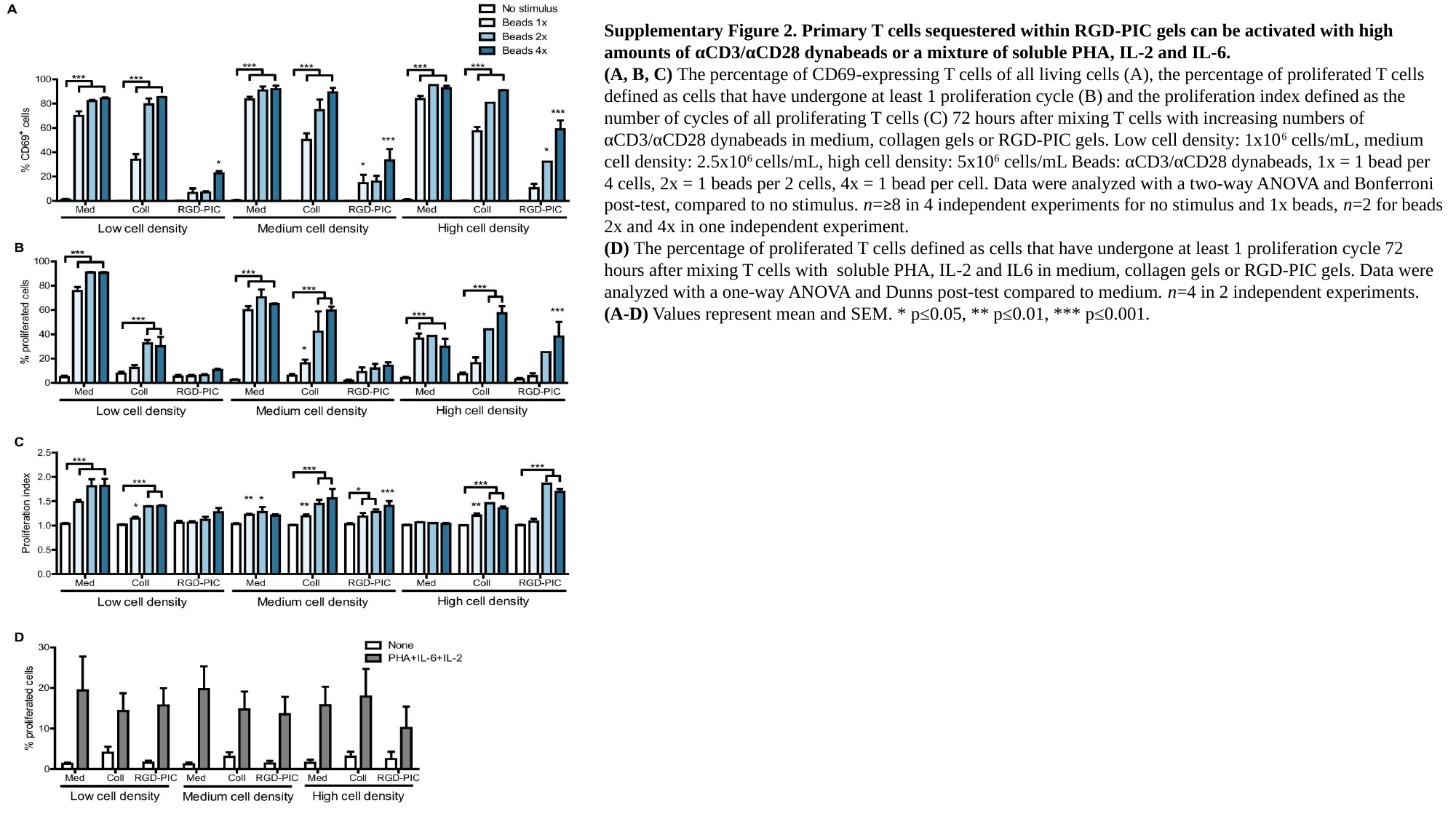

Supplementary Figure 2. Primary T cells sequestered within RGD-PIC gels can be activated with high amounts of αCD3/αCD28 dynabeads or a mixture of soluble PHA, IL-2 and IL-6.
(A, B, C) The percentage of CD69-expressing T cells of all living cells (A), the percentage of proliferated T cells defined as cells that have undergone at least 1 proliferation cycle (B) and the proliferation index defined as the number of cycles of all proliferating T cells (C) 72 hours after mixing T cells with increasing numbers of αCD3/αCD28 dynabeads in medium, collagen gels or RGD-PIC gels. Low cell density: 1x10^6^ cells/mL, medium cell density: 2.5x10^6^ cells/mL, high cell density: 5x10^6^ cells/mL Beads: αCD3/αCD28 dynabeads, 1x = 1 bead per 4 cells, 2x = 1 beads per 2 cells, 4x = 1 bead per cell. Data were analyzed with a two-way ANOVA and Bonferroni post-test, compared to no stimulus. n=≥8 in 4 independent experiments for no stimulus and 1x beads, n=2 for beads 2x and 4x in one independent experiment.
(D) The percentage of proliferated T cells defined as cells that have undergone at least 1 proliferation cycle 72 hours after mixing T cells with soluble PHA, IL-2 and IL6 in medium, collagen gels or RGD-PIC gels. Data were analyzed with a one-way ANOVA and Dunns post-test compared to medium. n=4 in 2 independent experiments.
(A-D) Values represent mean and SEM. * p≤0.05, ** p≤0.01, *** p≤0.001.

## Slide 3
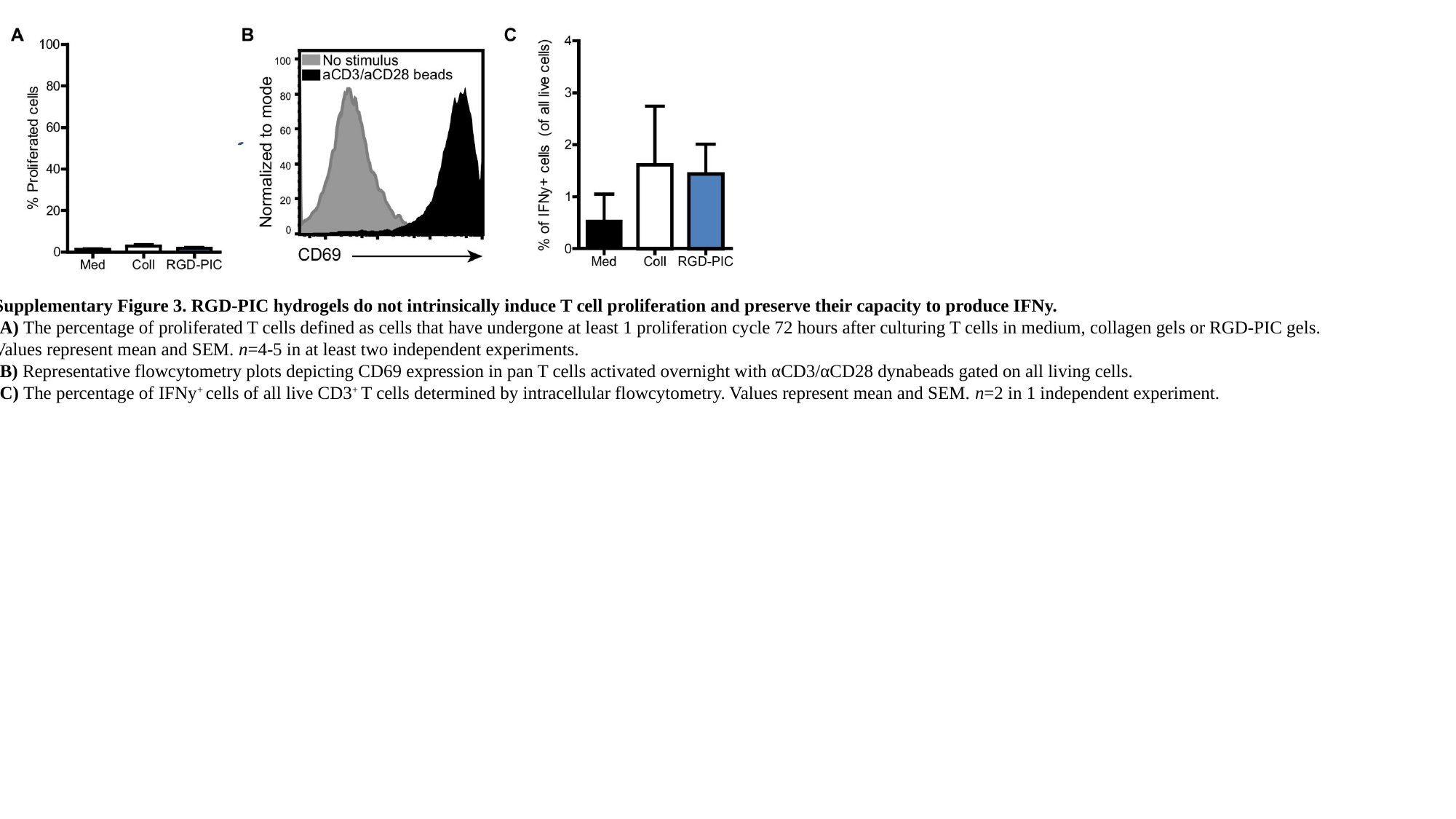

Supplementary Figure 3. RGD-PIC hydrogels do not intrinsically induce T cell proliferation and preserve their capacity to produce IFNy.
(A) The percentage of proliferated T cells defined as cells that have undergone at least 1 proliferation cycle 72 hours after culturing T cells in medium, collagen gels or RGD-PIC gels.
Values represent mean and SEM. n=4-5 in at least two independent experiments.
(B) Representative flowcytometry plots depicting CD69 expression in pan T cells activated overnight with αCD3/αCD28 dynabeads gated on all living cells.
(C) The percentage of IFNy+ cells of all live CD3+ T cells determined by intracellular flowcytometry. Values represent mean and SEM. n=2 in 1 independent experiment.

## Slide 4
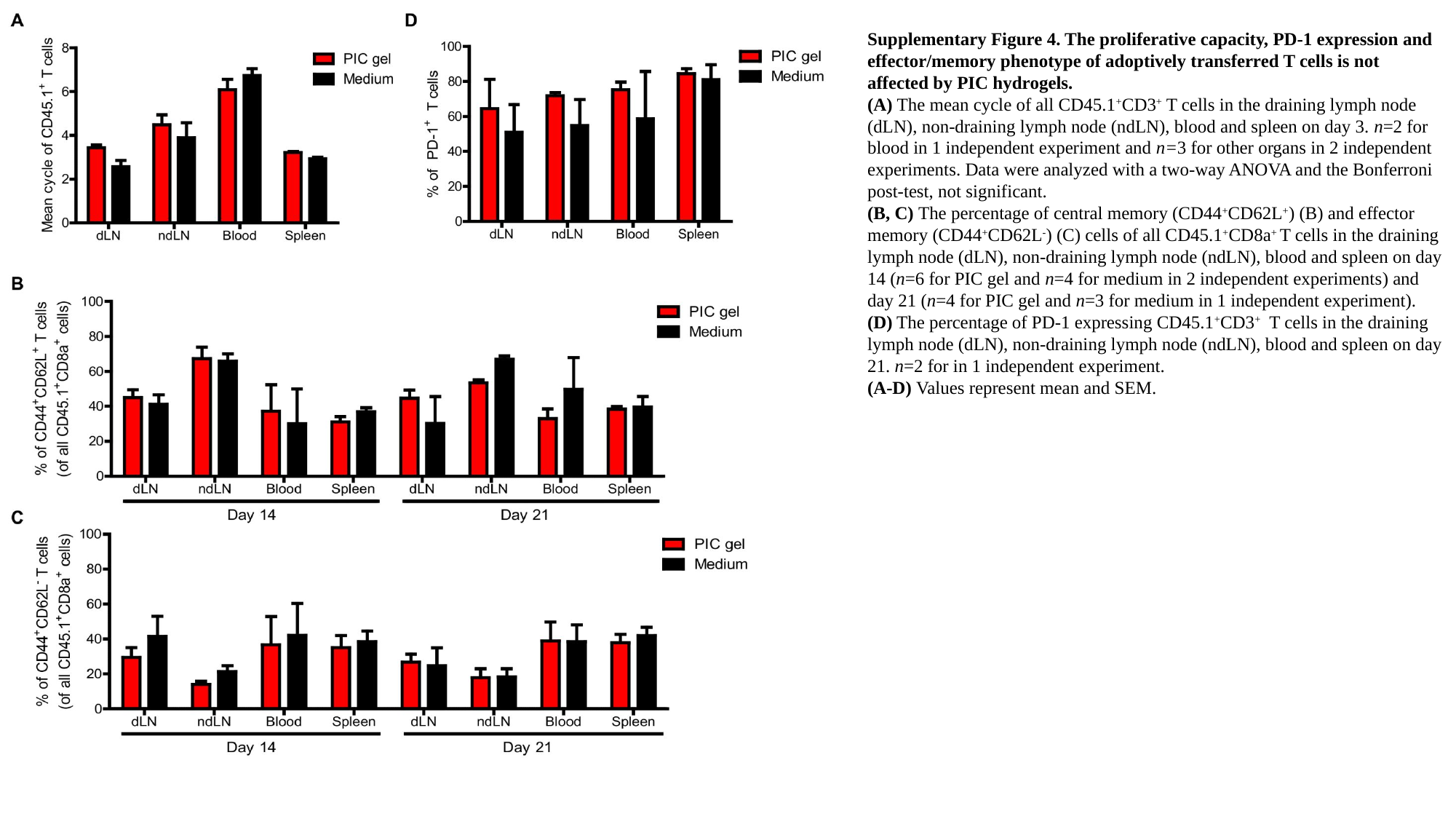

Supplementary Figure 4. The proliferative capacity, PD-1 expression and effector/memory phenotype of adoptively transferred T cells is not affected by PIC hydrogels.
(A) The mean cycle of all CD45.1+CD3+ T cells in the draining lymph node (dLN), non-draining lymph node (ndLN), blood and spleen on day 3. n=2 for blood in 1 independent experiment and n=3 for other organs in 2 independent experiments. Data were analyzed with a two-way ANOVA and the Bonferroni post-test, not significant.
(B, C) The percentage of central memory (CD44+CD62L+) (B) and effector memory (CD44+CD62L-) (C) cells of all CD45.1+CD8a+ T cells in the draining lymph node (dLN), non-draining lymph node (ndLN), blood and spleen on day 14 (n=6 for PIC gel and n=4 for medium in 2 independent experiments) and day 21 (n=4 for PIC gel and n=3 for medium in 1 independent experiment). (D) The percentage of PD-1 expressing CD45.1+CD3+ T cells in the draining lymph node (dLN), non-draining lymph node (ndLN), blood and spleen on day 21. n=2 for in 1 independent experiment.
(A-D) Values represent mean and SEM.
